# Hemoperfusion Using the Oxiris Membrane in Septic Shock Patients with Preserved Kidney Function: A Case Series

**DOI:** 10.3390/jcm14062113

**Published:** 2025-03-19

**Authors:** Darja Smirnova, Rihards Serzans, Mara Klibus, Valdis Liguts, Anna Lece, Andrejs Skesters, Gianluca Villa, Olegs Sabelnikovs

**Affiliations:** 1 Department of Anesthesiology, Intensive Care and Clinical Simulations, Riga Stradiņš University, LV-1007 Riga, Latvia; mara.klibus@rsu.lv (M.K.); olegs.sabelnikovs@rsu.lv (O.S.); 2 Department of Anesthesiology and Reanimatology, Pauls Stradiņš Clinical University Hospital, LV-1002 Riga, Latvia; rihards.serzans@rsu.edu.lv (R.S.); valdis.liguts@stradini.lv (V.L.); 3 Scientific Laboratory of Biochemistry, Institute of Occupational Safety and Environmental Health, Riga Stradinš University, LV-1007 Riga, Latvia; anna.lece@rsu.lv (A.L.); andrejs.skesters@rsu.lv (A.S.); 4 Department of Anesthesia and Intensive Care, Section of Oncological Anesthesia and Intensive Care, Careggi University Hospital, 50134 Florence, Italy; gianlucca.villa@unifi.it

**Keywords:** hemoperfusion, septic shock, endotoxin, Oxiris, immunoadsorption

## Abstract

**Background/Objectives**: Sepsis, a life-threatening condition caused by a dysregulated immune response to infection, is associated with high mortality. Endotoxin and cytokine overload play a crucial role in sepsis-induced organ dysfunction. The Oxiris^®^ membrane, traditionally used as a hemofilter for renal replacement therapy, has demonstrated the capacity to adsorb endotoxins and cytokines. This study investigates the clinical effect during hemoperfusion with the Oxiris^®^ membrane in patients with septic shock and preserved renal function. **Methods**: We present three adult patients with septic shock who were admitted to the intensive care unit with high vasopressor requirements and elevated inflammatory markers. As they were refractory to standard therapy and renal function was preserved, a 12-hour hemoperfusion session with an Oxiris^®^ membrane was initiated. Hemodynamic parameters, inflammatory biomarkers, and endotoxin concentrations were evaluated before, during, and after hemoperfusion treatment. **Results**: All patients demonstrated hemodynamic stabilization, with norepinephrine support reduced by 10.3% to 70.0%. Key inflammatory markers decreased significantly, including interleukin-6 (−41.6% to −94.0%), procalcitonin (−29.3% to −49.5%), and C-reactive protein (4.7% to −37.2%). Endotoxin concentrations decreased by 62.0% and 13.6% in two of the three patients. No adverse effects related to hemoperfusion were observed. **Conclusions**: Hemoperfusion with the Oxiris^®^ membrane effectively reduced vasopressor support, inflammatory markers, and endotoxin concentrations in patients with refractory septic shock. This approach may offer a novel strategy for early immune modulation in sepsis before renal dysfunction occurs. Further studies with larger cohorts are required to validate these findings and determine optimal treatment protocols.

## 1. Introduction

Sepsis is a life-threatening condition characterized by a dysregulated inflammatory response to infection, leading to organ dysfunction and high mortality [1]. The pathophysiology of bacterial sepsis is based on an exaggerated hyperinflammatory response triggered by endotoxins (the principal component of the outer membrane of Gram-negative bacteria), with subsequent uncontrolled release of pro- and anti-inflammatory cytokines. Studies show that higher endotoxin and cytokine concentrations in systemic circulation are responsible for higher mortality [2,3,4]. Therefore, extracorporeal techniques aimed at lowering the concentration of circulating inflammatory mediators may provide supportive care to maintain critical organ function and potentially improve the outcome of a septic patient. However, there are still many uncertainties about the most appropriate extracorporeal blood purification method, modality, initiation time, and duration of the therapy.

A novel blood purification device, the Oxiris^®^ hemofilter (GAMBRO Industries, Baxter, Deerfield, IL, USA), is made of a membrane with three different layers that allow the combination of renal support, endotoxin and cytokine removal, and local anticoagulation properties via heparin grafting in one device. The bulk of the hemofilter originates from a negatively charged acrylonitrile and sodium methallylsulfonate copolymer called the AN69 membrane, which provides the removal of both solutes and positively charged cytokines via convention and adsorption. The positively charged polyethyleneimine (PEI) layer enhances the hemocompatibility of the AN69 membrane by neutralizing the negative charges and, at the same time, enables the adsorption of negatively charged endotoxins. Oxiris^®^ has demonstrated effective adsorption of endotoxin and cytokines in vitro [5]. In addition, it has shown promising results in hemodynamic stabilization, reducing lactate levels, endotoxins, and cytokines and having a positive impact on mortality in patients with sepsis-associated acute kidney injury [6,7,8].

While the Oxiris^®^ membrane is designed as a hemofilter and is traditionally used during continuous renal replacement therapy (CRRT), its properties allow it to be used as a stand-alone modality for hemoperfusion to reduce the concentration of cytokines and endotoxins. In this context, the Oxiris^®^ membrane should be connected to the renal replacement machine (PrisMax or Prismaflex system) to perform hemoperfusion, and the modality “slow continuous ultrafiltration” without fluid withdrawal should be set up in the prescription settings, as stated in the official instructions for use of the distributor (revised version 2023-07-01). However, only a few studies are known to date that use Oxiris^®^ as a pure hemoperfusion device without renal function replacement [9,10].

Oxiris^®^ is essentially a hemofilter but has adsorption capacities that allow non-selective adsorption of endotoxins and cytokines. This poses a challenge for nomenclature, as Oxiris^®^ is not a conventional hemofilter but also does not meet the definition of a classical hemoadsorbent. Therefore, we will refer to the procedure as hemoperfusion in the following text, emphasizing the main reason for initiating the treatment—the removal of inflammatory mediators.

In our case series, we report the clinical changes during hemoperfusion with the Oxiris^®^ membrane in adult patients with septic shock refractory to conventional treatment. Our study shows a different view of early application of the Oxiris^®^ hemadsorption membrane in patients with septic shock before the hyperinflammatory response leads to loss of renal function. The novel approach in the hemoperfusion modality used in our treatment protocol, with a minimal total effluent flow rate and systemic heparin anticoagulation (thus excluding convective clearance), allowed a precise evaluation of the immune-adsorptive capability of the Oxiris^®^ membrane in reducing cytokines and endotoxins in vivo.

## 2. Materials and Methods

### 2.1. Description of the Study Population

**Figure 1:**
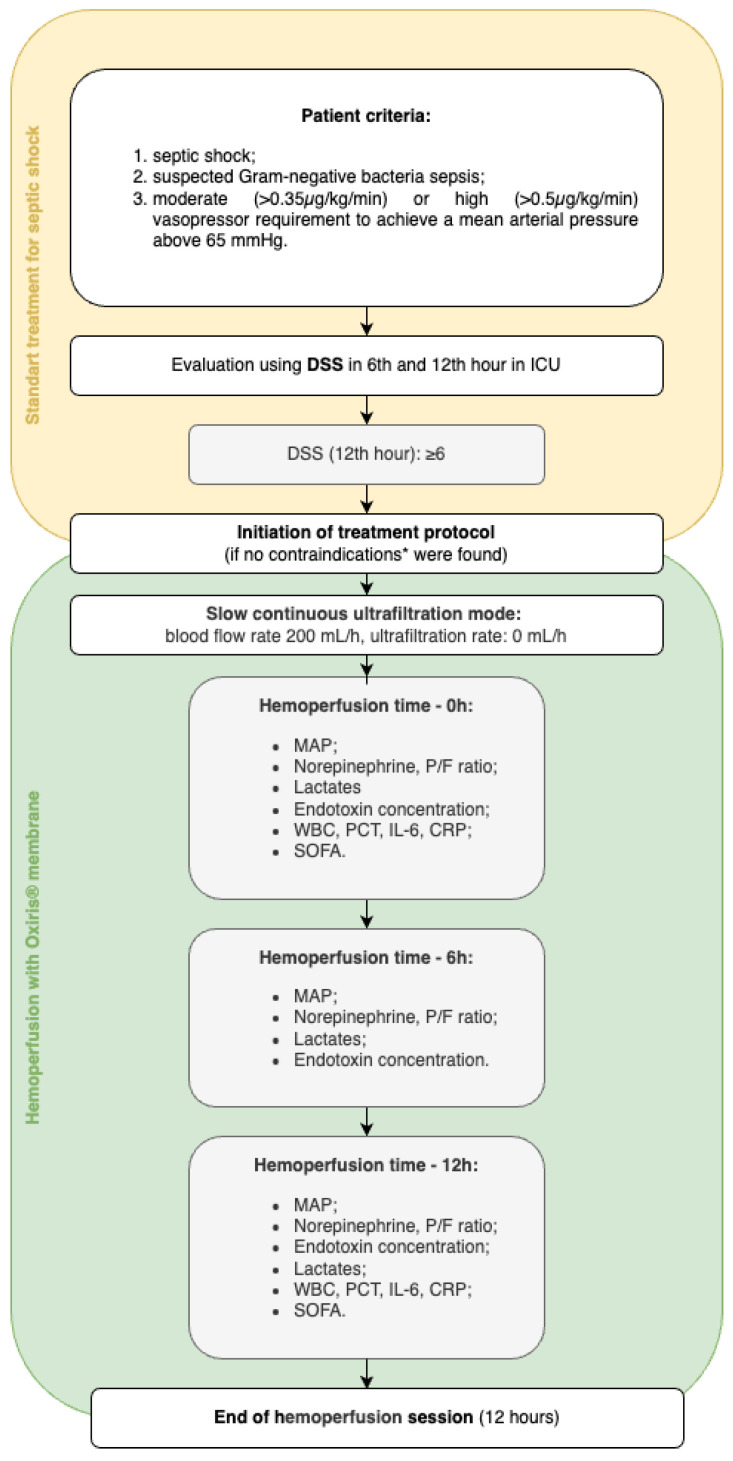
Research methodology. * Contraindications for treatment protocol initiation were severe thrombocytopenia, contraindication to anticoagulation with heparin, or inability to provide informed consent. Abbreviations: CRP—C-reactive protein; DSS—Dynamic Score System; ICU—intensive care unit; IL-6—interleukin-6; MAP—mean arterial pressure; P/F—ratio of partial pressure of oxygen in arterial blood to the fraction of inspiratory oxygen concentration; PCT—procalcitonin; SOFA—Sequential Organ Failure Assessment; WBC—white blood cell.

In this case series, three adult patients with septic shock were admitted to the intensive care unit (ICU) in the tertiary care center in Pauls Stradiņš Clinical University Hospital in Riga, Latvia, and received hemoperfusion with an Oxiris^®^ membrane. The decision to initiate hemoperfusion was made if patients met the following criteria (Figure 1): septic shock (based on the Third International Consensus definition for Sepsis and Septic shock (Sepsis-3) [1]) with moderate (≥0.35 µg/kg/min) or high (≥0.50 µg/kg/min) norepinephrine requirement to achieve a mean arterial pressure above 65 mmHg, suspected Gram-negative bacteria with elevated inflammatory markers, and worsening clinical course despite standard treatment for septic shock according to the Surviving Sepsis Campaign guidelines [11]. Worsening of the clinical course was defined as an increase in Dynamic Score System (DSS) points during the first 12 h of ICU admission, reaching at least 6 points. Patients in whom any of the following contraindications to treatment were suspected—severe thrombocytopenia, contraindication to anticoagulation with heparin, or inability to provide informed consent—were excluded from further analysis and were provided with the standard treatment for severe septic shock.

### 2.2. Description of the Treatment Protocol and Technical Considerations for Hemoperfusion

All patients admitted to the ICU received the standard protocol for the treatment of septic shock: broad-spectrum antibiotics covering both Gram-negative and -positive flora, sufficient fluid resuscitation (30 mL/kg of balanced crystalloid for the first three hours and subsequent fluid according to clinical need), vasopressor support, and surgery to control the source of infection if required by the primary diagnosis. The dynamic changes in vasopressor and lactate levels in the first twelve hours after ICU admission were evaluated, and if the pathologic changes were scored at 6 or higher on the DSS, a situation refractory to standard therapy was defined and the patients were eligible for Oxiris^®^ hemoperfusion (Figure 1).

Under ultrasound guidance, a 13 French central venous catheter was placed in the right internal jugular vein, and the Oxiris set was connected to the PrisMax continuous renal replacement therapy (CRRT) system to perform a hemoperfusion session. Systemic heparinization was chosen as an anticoagulation strategy, and the following parameters were prescribed: blood flow rate of 200 mL/hour, and replacement flow rate, dialysate flow rate, and net ultrafiltration rate of 0 mL/hour. For this purpose, the technical modality “slow continuous ultrafiltration” was set on the CRRT machine, as recommended in the manufacturer’s instructions for use. Since the Oxiris^®^ filter is a porous membrane, its permeability could theoretically allow a minimal amount of fluid to pass through, especially under conditions of high blood flow and the adsorptive properties of the membrane, but this is not clinically significant. To reflect this more accurately, we refer to the approach as a “minimal effluent dose”.

Since hemoperfusion therapy was initiated early in septic shock patients with preserved diuresis, net ultrafiltration was not included in the treatment protocol. However, intensivists were permitted to remove fluid when clinically indicated. The duration of each hemoperfusion session was 12 h, after which it was discontinued. All included patients received at least one treatment session. One of the patients received a second 12-hour hemoperfusion session. The decision to repeat the hemoperfusion session was based on the clinical course of septic shock in the relevant case—clinical condition was improving (reduction in norepinephrine support and lactate levels) during and after the first session, seemingly as a positive effect of hemoperfusion, but failed to achieve a satisfactory state, still needing high norepinephrine support (≥0.5 µg/kg/min) and preserving high inflammatory markers.

### 2.3. Data Collection and Analysis

The data collection included information on patients’ data (age, gender, body mass index (BMI)); the main diagnosis prompting ICU admission; and pre-existing comorbidities, source control interventions, and bacterial growth in the blood. The data required for calculating the DSS score were collected, and the DSS score was determined twice within the first twelve hours after admission to the intensive care unit. Alterations in infection markers (white blood cells (WBCs), procalcitonin (PCT), interleukin-6 (IL-6), C-reactive protein (CRP), lactate (Lac)) and severity, as determined by Sequential Organ Failure Assessment (SOFA) scores, were analyzed before and after hemoperfusion. The amount of norepinephrine (NE), ratio of partial pressure of oxygen in arterial blood to the fraction of inspiratory oxygen concentration (P/F), and vital signs were analyzed during the hemoperfusion.

In addition, lactate levels and endotoxin concentrations were measured immediately before (0 h) and 6 and 12 h after the start of the hemoperfusion with Oxiris^®^. Endotoxin concentration was assessed using an enzyme-linked immunosorbent assay (ELISA) for the quantitative detection of Gram-negative endotoxins in human samples (Appendix A). Blood samples for endotoxin concentration measurement were collected from an extracorporeal circuit arterial line into a heparinized vial coated with ethylene-tetra-acetic acid (EDTA) and immediately centrifuged, and the plasma was stored at −80 °C for up to one month until analyses were performed in the Riga Stradiņš University Scientific Laboratory of Biochemistry.

The rate of complications possibly related to hemoperfusion was also documented.

Informal consent was obtained from all included patients or from a person with decision-making responsibility prior to data collection (including information about the methods used, data protection measures, conditions of participation, the possibility to refuse the use of their data, and the further use of their data).

### 2.4. Statistical Analysis

Descriptive statistics for participant characteristics and blood test results are reported. The analysis focused on absolute and relative changes in key clinical and biochemical parameters to evaluate the effects of the hemoperfusion session. The primary outcomes were a reduction in norepinephrine support, improvements in hemodynamic stability (MAP, respiratory stability (P/F ratio)), and changes in biomarkers (WBC, CRP, PCT, IL-6, Lac, and endotoxin concentration) after hemoperfusion.

Absolute differences before and after the hemoperfusion protocol in norepinephrine support, MAP, P/F ratio, and biomarkers were calculated as follows: [Absolute change] = [Post-treatment value] − [Pre-treatment value]. Percentage changes (%) for each parameter were calculated as follows: [Relative change] = ([Post-treatment value] − [Pre-treatment value])/Pre-treatment value × 100%.

Given the nature of the study as a case series with only three patients, statistical significance was not assessed. Instead, the analysis focused on clinically important changes in key hemodynamic and biochemical parameters.

## 3. Case Presentation and Description

In our case series, we are presenting three cases of septic shock in which the previously mentioned treatment protocol was initiated. All of these patients survived until the hospital discharge for further rehabilitation and did not require further lung or kidney support. A short overview of the cases described is shown in Table 1.

### 3.1. Clinical Case 1 (Patient A)

A 61-year-old male was admitted to the hospital with complaints of shortness of breath and radiating pain from the left side of the thorax to the left hand. These symptoms had progressively worsened over the previous three days. Blood analysis revealed high levels of inflammatory markers. A computed tomography scan of the chest confirmed right-sided pleurisy, and the patient was transferred to the pulmonology department. Under direct ultrasonographic guidance, a surgical drain was inserted, and pleural fluid culture was obtained for bacteriological analysis.

Empirical treatment with amoxicillin/clavulanic acid was initiated, but this was soon switched to ampicillin/sulbactam and colistin when blood cultures identified *Acinetobacter baumannii*. On the 11th intrahospital day, the patient experienced first-time generalized seizures and increasing shortness of breath with desaturation; consequently, oxygen support via a high-flow nasal cannula was started. The following day, a video-assisted thoracoscopy was performed. Despite these interventions, respiratory failure and hemodynamic instability progressed and the patient was transferred to the ICU for initiation of invasive mechanical lung ventilation and continuation of hemodynamic support.

During the first six hours in the ICU, the patient scored four points on the DSS. By the 12th hour, the DSS score had increased to six points. The hemodynamic instability had progressed, and norepinephrine infusion had been increased to 0.35 µg/kg/min.

As the patient met the inclusion criteria, the baseline concentrations of endotoxins and inflammatory markers in the blood were determined, and hemoperfusion with the Oxiris^®^ membrane based on the previously mentioned parameters was initiated. After 12 h of hemoperfusion, stabilization of the general condition was observed (Table 2 and Table 3): endotoxin concentrations decreased, inflammatory marker support had been reduced, SOFA score decreased, and respiratory function slightly improved. No complications related to hemoperfusion were observed during the treatment, and the patient had preserved a spontaneous diuresis of 2 L, corresponding to stage I acute kidney injury according to the Kidney Disease: Improving Global Outcomes (KDIGO 2012) classification [12]. Norepinephrine support gradually decreased until being discontinued completely on the sixth day in the ICU.

After twenty-two days in the ICU, the patient was transferred back to the pulmonology department and 11 days later was completely weaned off the ventilator and subsequently discharged.

### 3.2. Clinical Case 2 (Patient B)

A 53-year-old female was transferred from a municipal secondary care hospital with bilateral hydropneumothorax and a suspected diagnosis of esophageal perforation and mediastinitis. The patient had previously been diagnosed with phenylketonuria and severe mental disability. Computed tomography with oral contrast revealed a defect in the frontal wall of the lower third of the esophagus, with spreading of contrast medium into the pleural space.

The following day, esophageal stenting and video-assisted thoracoscopy with surgical drainage were performed, and afterwards, the patient was transferred to the ICU with septic shock for hemodynamic and respiratory support and continuation of antibacterial therapy with piperacillin/tazobactam. Initial blood bacteriology results were sterile; however, intraoperatively taken pleural fluid analysis was positive for *Streptococcus anginosus*. In the first 6 h in the ICU, the DSS score was four points. At 12 h, the DSS score had increased to six points. A hemoperfusion session was initiated. At the end of hemoperfusion, the patient had stabilized (Table 2 and Table 3). Norepinephrine support was gradually reduced, accompanied by an increase in the P/F ratio and a slight decrease in endotoxin concentration. Inflammatory markers also improved. No hemoperfusion-related complications were observed, and spontaneous diuresis during the procedure was 1.5 L.

On the fourth day of hospitalization, the patient was successfully weaned off mechanical ventilation. The following day, norepinephrine support was discontinued, and the patient was transferred to the pulmonology department for further treatment. The patient was discharged 29 days later.

### 3.3. Clinical Case 3 (Patient C)

A 73-year-old female was admitted to the hospital with a primary complaint of constipation that had persisted for the previous three days. Her past medical history included a laparoscopic cholecystectomy three years earlier, chronic autoimmune thyroiditis, non-Hodgkin’s lymphoma, a spontaneous intracerebral hemorrhage, diverticulosis, and dementia. 

Upon admission, diagnostic imaging revealed small intestinal ileus, gastric stasis, acute bilateral lobar (aspiration) pneumonia, and an incarcerated hiatal hernia. Given the critical findings, an urgent surgical intervention was performed, which included endotracheal intubation, herniotomy, small intestine resection, and enteroenteric anastomosis.

During the perioperative period, the patient’s condition deteriorated, leading to septic shock and necessitating transfer to the ICU for further treatment. Bacterial cultures taken from the tracheal aspirate on admission were positive for methicillin-susceptible *Staphylococcus aureus* (MSSA) and *Escherichia coli*, and treatment with piperacillin–tazobactam was continued. Over the following 12 h in the ICU, the patient’s clinical condition worsened: the Dynamic Score System (DSS) score rose from six points at the sixth ICU hour to seven points, and hemodynamic instability and respiratory support requirements progressed (Table 3). Consequently, the protocol was initiated, and after 12 h, hemoperfusion was discontinued as per the protocol. By the end of the session (Table 2 and Table 3), the patient’s overall clinical status had slightly improved, with a reduction in norepinephrine support and notable improvements in lung function. Additionally, most inflammatory markers showed a significant decrease.

However, as inflammatory markers remained persistently elevated over the next 12 h (Table 2 and Table 3) and no significant clinical improvement was observed aside from a slight reduction in norepinephrine infusion, a second hemoperfusion session was initiated. Following this session, hemodynamic stability improved further, alongside enhancements in respiratory function. Additionally, overall immune response markers and sepsis biomarkers exhibited a downward trend, indicating a positive therapeutic effect.

No complications related to hemoperfusion were observed during any of the treatment sessions, and the patient had preserved a spontaneous diuresis of 0.4 L (classified as stage I acute kidney injury per the KDIGO 2012 criteria [12]) and 4.4 L, respectively. Vasopressor support ended on the fifth day of hospitalization, and the patient was weaned off mechanical ventilation on the eighth day. Two days later, she was transferred to the pulmonology department and subsequently transferred back to the municipal secondary care hospital on day 28.

## 4. Discussion

### 4.1. Summary of Main Findings

In this case series, we report a new treatment approach for hemoperfusion therapy with the Oxiris^®^ membrane in patients with septic shock before hyperinflammation leads to loss of renal function. Hemoperfusion was performed in patients with residual spontaneous diuresis, with the main aim being the reduction in inflammatory mediators.

All patients received standard treatment for septic shock, including fluid resuscitation, antimicrobial therapy, and surgical source control, according to established protocols prior to the initiation of hemoperfusion with the Oxiris^®^ hemofilter. Source control interventions were performed in a timely manner and in accordance with clinical guidelines. Subsequent clinical and imaging examinations revealed no indications for additional surgical interventions, confirming the effectiveness of initial source control in all cases. In addition, all patients in our case series received broad-spectrum antibiotics targeting the isolated pathogen responsible for septic shock in a timely manner, ensuring complete compliance with sepsis treatment guidelines prior to the initiation of hemoperfusion.

The Dynamic Score System (DSS) was used with a six-point threshold as an indicator for initiating treatment. The rationale for this methodology is based on the scoring system introduced by Kogelmann et al. in 2021 [13], which suggests that a score of 6–8 points represents the optimal window for initiating immunoadsorption therapy with CytoSorb. This range strikes a balance between the benefits of early intervention and the risk of overtreatment. Although the DSS was originally developed for CytoSorb, its application to Oxiris is rational, given the absence of a similar scoring system for this therapy.

We found that 12 h of early initiated hemoperfusion with Oxiris^®^ was associated with a markable reduction in inflammatory mediators such as IL-6 and inflammatory markers (such as CRP, PCT, and WBCs) and an improvement in hemodynamic status (related to reduction in norepinephrine support). In addition, an improvement in organ function score (SOFA) was also observed when comparing pre- and post-treatment values. No complications possibly related to hemoperfusion (severe reduction in thrombocytes, electrolyte level depletion, central catheter infections, or hemothorax) were documented. All patients did not require additional renal replacement therapy during treatment and were discharged from the ICU after a mean of 12 days without the need for hemodynamic, renal, or pulmonary support.

The baseline endotoxin concentration was significantly increased in all patients, averaging at 1.18 EU/mL, and two patients (Patient A and Patient B) showed a reduction in endotoxin levels after a 12-hour treatment session (by 62.0% and 13.6%, respectively). Patient C maintained a high endotoxin concentration, which was reflected in the relatively high norepinephrine support needed after the first hemoperfusion session, and a second treatment session was required. Although significant clinical and laboratory improvements were observed during the hemoperfusion session in Patient C (related to the reduction in norepinephrine support and inflammatory markers and improvement in respiratory status), the post-treatment endotoxin concentration remained the same as the pre-treatment concentration. This finding appears to be related to an overproduction of endotoxins rather than the inability of the membrane to adsorb endotoxins through the positively charged PEI layer. It is known that many factors besides hemoadsorption contribute to endotoxin load in the blood compartment, as endotoxin concentration is the result of an imbalance between endotoxin production and elimination [14]. Endotoxin production depends mainly on the bacterial load, the characteristics of the bacterial species, and the bacterial degradation caused by the immune system or antibacterial therapy, with subsequent release of endotoxins into the bloodstream. Furthermore, endotoxin production in vivo is not a single pulse, but rather a constant seeding from bacterial colonies. This could theoretically explain our finding of an increased endotoxin concentration at the 6-hour time point in the blood samples of Patient C. The elimination of endotoxins is mainly related to endotoxin degradation by the host’s immune system, spontaneous degradation of endotoxins, and adsorption of endotoxins by a suitable hemadsorption device, such as the PEI layer in the case of Oxiris^®^.

### 4.2. Reflection on Previous Studies

Although several types of extracorporeal blood purification devices are in clinical use to remove excess endotoxins, Oxiris^®^ is the only device designed to remove both cytokines and endotoxins from circulation. In addition, its cost is relatively low compared to other direct endotoxin hemoadsorbents [15], it is easy to use with continuous renal replacement therapy machines, and its endotoxin removal capacity is similarly effective [5]. Our decision to start hemoperfusion with the Oxiris^®^ membrane was driven by the availability of the hemoperfusion devices for endotoxin removal in our clinical center.

Oxiris^®^ is traditionally used as a hemofilter in renal replacement therapy and, according to a recently published meta-analysis [6,8], could provide beneficial effects on the clinical course of patients with septic shock. However, the timing of the initiation of Oxiris^®^ treatment remains controversial. In the absence of strict guidelines, the clinical use of Oxiris^®^ depends on the decision at institutional level. According to the Asia-Pacific expert consensus published in 2021, Oxiris^®^ can be considered in patients with sepsis also before acute kidney injury (AKI) develops, based on several key clinical factors such as severe hemodynamic instability, microcirculatory and organ dysfunction [16]. Once patients meet the indication for treatment, CRRT with Oxiris should be initiated as soon as possible.

While Oxiris^®^ is designed as a hemofilter, its chemical characteristics allow the use of its immuno-adsorptive properties. In the context of hemoperfusion, the SCUF technical modality with no fluid removal prescription is recommended. However, only a few studies are known to date that used Oxiris^®^ as a hemoperfusion device. In the recent SIRAKI02 randomized controlled trial investigating the ability of Oxiris^®^ to reduce the incidence of AKI in patients undergoing non-emergent cardiac surgery, the Oxiris^®^ membrane was connected to the extracorporeal circuit of the cardiopulmonary bypass in SCUF modality with an ultrafiltration rate of 0 mL/h and a blood flow rate of 200–300 mL/min [10]. In addition to the primary endpoint results, significant reductions in tumor necrosis factor α and interleukin-8 plasma concentrations during the cardiopulmonary bypass were found in the Oxiris^®^ group compared to the control group. Another group of researchers from Germany recently published an experimental animal study in which the clinical efficacy of Oxiris^®^ in reducing cytokines after endotoxin infusions was investigated using the pumpless extracorporeal hemadsorption technique [9]. No differences were observed between the Oxiris^®^ and control groups in terms of cytokine reduction during the six-hour therapy. However, since the study methodology required a single infusion of the endotoxins with subsequent spontaneous release of interleukins, the endotoxin load might have been insufficient to achieve an imbalance between the production and removal of inflammatory mediators. Therefore, the lack of difference in the concentration of interleukins after 6 h of hemadsorption between the two groups may have been due to adequate spontaneous degradation of mediators by the host immune system rather than immunoadsorption of the membrane.

In our hemoperfusion treatment protocol, the chosen technical settings on the CRRT machine (SCUF modality with minimal effluent dose) and anticoagulation strategy offered the possibility to exclude the effect of elimination of inflammatory markers by convection, thus allowing us to evaluate the exact immuno-absorptive capacity of the membrane. This provided new insights to better understand the hemadsorption properties of the Oxiris^®^ membrane in in vivo situations. The decision to use a relatively short treatment duration (twelve hours) was made to reduce the risk of a membrane saturation phenomenon, i.e., the time during which the membrane exhausts its own capacity to absorb cytokines and endotoxins. Although the saturation limit of the Oxiris^®^ membrane is still under discussion, elective change prior to 12 h is recommended to achieve optimal immunoadsorption [17,18].

Finally, the modality chosen in our treatment protocol might have a lower cost compared to continuous venovenous hemofiltration (CVVH) with Oxiris^®^, with similar efficacy during the first 12 h of treatment, as reported in our previous research [19] (unpublished data), mainly because there is no additional cost for replacement fluid. In this prospective randomized study, the novel hemoperfusion modality was compared with the conventional CVVH modality. Both groups showed a significant reduction in vasopressor support in the early phase of treatment. The group of patients treated with isolated hemoperfusion showed a significant reduction in endotoxins compared to the conventional group, although the basal endotoxin concentration was higher.

### 4.3. Strength and Limitation

Our study provides novel insights into the exact immunoadsorption properties of the Oxiris^®^ membrane. Another strength of our study is the detection of endotoxin concentrations and the implementation of a new diagnostic approach using the Dynamic Score System to dynamically assess the severity of the clinical course in patients with septic shock. We believe that this dynamic assessment of refractory septic shock could help clinicians decide on the optimal time to initiate hemoperfusion. However, there are some limitations that could be considered in further studies. First, the main limitation of this study is the relatively small number of participants. Secondly, we lacked a control group to clarify whether the decrease in endotoxin concentration was related to the hemadsorption properties of the membrane or the spontaneous degradation of endotoxins. Future studies should include a control group with a conventional treatment approach. Expanding the sample size and comparing with a control group would enhance the validity of the study. Thirdly, we used ELISA assays instead of the endotoxin activity assay (EAA) in our case studies. Therefore, the endotoxin concentration results were shifted in time, limiting the comprehensive real-time understanding of the treatment effect. The use of rapid qualitative tests to detect endotoxins, such as the EAA, would simplify the inclusion criteria and initiation of the treatment protocol. However, this test is not widely used in our region. Fourthly, our treatment protocol utilized a hemoperfusion modality with a negligible total effluent flow rate under systemic heparinization (technically set as the SCUF modality in the CRRT machine), excluding both diffusive and convective clearance as well as fluid removal. While this approach enables the evaluation of the pure immunoadsorption properties of the Oxiris^®^ membrane, our study did not quantify the impact of convective clearance on the cytokine removal efficiency of the AN69 layer. Fifthly, in our study, we replaced the Oxiris cartridge after 12 h to mitigate the potential impact of membrane saturation and to ensure that inflammatory mediator clearance was not compromised by adsorptive exhaustion. However, it remains uncertain to what extent the membrane reaches full saturation at this point or whether its efficacy persists beyond this time frame. Future studies evaluating real-time adsorption kinetics and prolonged use may provide further insight into this aspect. Finally, in our study, antibiotic clearance was not monitored during the hemoperfusion session. This raises concern about the unintended removal of antimicrobials, which could potentially compromise treatment efficacy. Future studies should account for this effect and implement appropriate strategies to optimize antibiotic dosing and ensure optimal patient outcomes.

## 5. Conclusions

In conclusion, early hemoperfusion with the Oxiris^®^ membrane was used to manage three patients with septic shock who did not respond to conventional therapy. The treatment resulted in significant clearance of inflammatory mediators such as interleukin-6 and endotoxins. This reduction was associated with changes in hemodynamics, including a decreased need for norepinephrine to maintain mean arterial pressure, as well as the preservation of organ function. While these findings suggest potential benefits, further studies with larger sample sizes and a control group are needed to evaluate the efficacy and safety of this treatment before it can be routinely implemented in clinical practice.

## Figures and Tables

**Table 1:** Patient characteristics and hospitalization data.

Patient	Gender	Age, Years	BMI, kg/m²	Sepsis Cause	Bacteriology Result	Intrahospital Days	ICU Days	Norepinephrine Days
A	Male	61	24.5	Pleural empyema	*A. baumannii* ^1^	76	23	7
B	Female	53	17.9	Mediastinitis	*S. anginosus* ^2^	34	4	4
C	Female	73	20.2	Pneumonia	*MSSA*, *E. coli* ^3^	28	10	5

Available bacteriology results at the moment of protocol initiation are shown: ^1^—blood bacteriology results positive for *Acinetobacter baumannii*; ^2^—pleural fluid bacteriology results positive for *Streptococcus anginosus*; ^3^—tracheal aspirate bacteriology results positive for methicillin-susceptible *Staphylococcus aureus* and *Escherichia coli*. Abbreviations: BMI—body mass index; ICU—intensive care unit.

**Table 2:** Immune responses, sepsis biomarkers, and SOFA dynamic changes before and after hemoperfusion.

Patient	Marker/Parameter	Hemoperfusion Time		Absolute Change, Δ	Relative Change, %
		0 h	12 h		
A	WBC, ×10^9^/L	15.3	11.8	−3.5	−22.88
	CRP, mg/L	587.95	369.39	−218.56	−37.17
	PCT, ng/mL	11.09	6.48	−4.61	−41.57
	IL-6, pg/mL	398.6	232.9	−165.7	−41.57
	SOFA	10	9	-	-
B	WBC, ×10^9^/L	10.2	8.1	−2.1	−20.59
	CRP, mg/L	411.55	370.04	−41.51	−10.09
	PCT, ng/mL	22.17	15.68	−6.49	−29.27
	IL-6, pg/mL	162.2	34.5	−127.7	−78.73
	SOFA	7	5	-	-
C ^1^	WBC, ×10^9^/L	9.4	14.0	+4.6	+48.94
	CRP, mg/L	288.62	302.12	+13.5	+4.68
	PCT, ng/mL	359.59	181.47	−178.12	−49.53
	IL-6, pg/mL	1270.1	76.8	−1193.3	−93.95
	SOFA	10	9	-	-
C ^2^	WBC, ×10^9^/L	14.0	10.1	−3.9	−27.86
	CRP, mg/L	301.75	257.75	−44.00	−14.58
	PCT, ng/mL	122.11	68.76	−53.35	−43.69
	IL-6, pg/mL	133.3	69.5	−63.8	−47.86
	SOFA	10	9	-	-

^1^—first filter; ^2^—second filter. Abbreviations: CRP—C-reactive protein; IL-6—interleukin-6; PCT—procalcitonin; SOFA—Sequential Organ Failure Assessment; WBC—white blood cell.

**Table 3:** Hemodynamic, respiratory, endotoxin, and lactate changes before, during, and after hemoperfusion.

Patient	Parameter	Hemoperfusion Time			Absolute Change, Δ	Relative Change, %
		0 h	6 h	12 h		
A	NE, µg/kg/min	0.35	0.27	0.14	−0.21	−56.25
	MAP, mmHg	83	98	81	−2	−2.41
	P/F ratio	206.7	247.3	228.0	+21.3	+10.30
	Lac, mmol/L	1.2	1.0	1.3	+0.1	+8.33
	ET, EU/mL	0.50	0.48	0.19	−0.31	−62.00
B	NE, µg/kg/min	0.5	0.48	0.43	−0.07	−14.00
	MAP, mmHg	72	72	81	+9	+12.50
	P/F ratio	387.5	395.2	428.6	+41.1	+10.61
	Lac, mmol/L	1.8	1.8	1.2	−0.6	−33.33
	ET, EU/mL	1.55	1.52	1.34	−0.21	−13.55
C ^1^	NE, µg/kg/min	0.97	0.97	0.87	−0.1	−10.31
	MAP, mmHg	73	72	94	+11	+15.07
	P/F ratio	268.0	201.0	332.0	+64	+23.88
	Lac, mmol/L	2.6	2.7	3.0	+0.4	+15.38
	ET, EU/mL	1.50	1.70	1.60	+0.1	+6.67
C ^2^	NE, µg/kg/min	0.50	0.19	0.15	−0.35	−70.00
	MAP, mmHg	95	98	92	−3	−3.16
	P/F ratio	357.1	306.7	363.3	+6.20	+1.74
	Lac, mmol/L	2.1	3.1	2.3	+0.2	+9.52
	ET, EU/mL	1.55	1.71	1.50	−0.05	−3.22

^1^—first filter; ^2^—second filter. Abbreviations: ET—endotoxin; Lac—lactate; MAP—mean arterial pressure; NE—norepinephrine; P/F—ratio of partial pressure of oxygen in arterial blood to the fraction of inspiratory oxygen concentration.

## Data Availability

The original contributions presented in this study are included in the article; further inquiries can be directed to the corresponding author.

## Abbreviations

The following abbreviations are used in this manuscript:

AKI	Acute kidney injury
BMI	Body mass index
CRP	C-reactive protein
CRRT	Continuous renal replacement therapy
CVVH	Continuous venovenous hemofiltration
DSS	Dynamic Score System
EAA	Endotoxin activity assay
EDTA	Ethylene-tetra-acetic acid
ELISA	Enzyme-linked immunosorbent assay
ET	Endotoxin
ICU	Intensive care unit
IL-6	Interleukin-6
Lac	Lactate
MAP	Mean arterial pressure
MSSA	Methicillin-susceptible *Staphylococcus aureus*
NE	Norepinephrine
OD	Optical density
P/F	Ratio of partial pressure of oxygen in arterial blood to the fraction of inspiratory oxygen concentration
PCT	Procalcitonin
PEI	Polyethyleneimine
SCUF	Slow continuous ultrafiltration
SOFA	Sequential Organ Failure Assessment
WBC	White blood cell

## Appendix A

Blood samples for endotoxin concentration measurement were collected from an extracorporeal circuit arterial line into a heparinized vial coated with EDTA and immediately centrifuged for 15 min at room temperature. Plasma samples were stored at −80 °C for up to one month until analyses were performed in the Riga Stradiņš University Scientific Laboratory of Biochemistry.

Endotoxin concentration was measured using a quantitative colorimetric enzyme-linked immunosorbent assay (ELISA) kit. This kit is specifically designed for detecting Gram-negative endotoxins in plasma samples, with a detection range of 100–2500 pg/mL (equivalent to 1–25 EU/mL) and a sensitivity of ≤0.005 EU/mL. The plasma samples were brought to room temperature (18–25 °C) without additional heating and diluted 1:10 in an endotoxin-free diluent before performing the assay. The assay procedure, including steps such as the addition of detection reagents, TMB substrate, stop solution, incubation times, and temperature settings, was performed strictly according to the manufacturer’s instructions (available at: LSBio ELISA Kit Instructions https://www.lsbio.com/elisakits/manualpdf/ls-f15272.pdf (accessed on 6 February 2025). The optical density (OD) of each diluted sample and control was measured immediately using a microplate reader (Tecan GmbH, Austria, Grödig) set to 450 nm. The measured OD values were then converted to endotoxin concentrations (EU/mL). The final endotoxin concentrations (reported in this study) in the plasma samples were calculated using the following formula: [Endotoxin concentration in the plasma sample] = [Determined concentration in the diluted plasma sample] × [dilution factor (11)].
